# Optimization of Engineered Production of the Glucoraphanin Precursor Dihomomethionine in *Nicotiana benthamiana*

**DOI:** 10.3389/fbioe.2016.00014

**Published:** 2016-02-16

**Authors:** Christoph Crocoll, Nadia Mirza, Michael Reichelt, Jonathan Gershenzon, Barbara Ann Halkier

**Affiliations:** ^1^DNRF Center DynaMo, Department of Plant and Environmental Sciences, Faculty of Science, University of Copenhagen, Frederiksberg, Denmark; ^2^Copenhagen Plant Science Center, Department of Plant and Environmental Sciences, Faculty of Science, University of Copenhagen, Frederiksberg, Denmark; ^3^Department of Biochemistry, Max Planck Institute for Chemical Ecology, Jena, Germany

**Keywords:** dihomomethionine, glucoraphanin, glucosinolates, metabolic engineering, *Nicotiana benthamiana*

## Abstract

Glucosinolates are natural products characteristic of the Brassicales order, which include vegetables such as cabbages and the model plant *Arabidopsis thaliana*. Glucoraphanin is the major glucosinolate in broccoli and associated with the health-promoting effects of broccoli consumption. Toward our goal of creating a rich source of glucoraphanin for dietary supplements, we have previously reported the feasibility of engineering glucoraphanin in *Nicotiana benthamiana* through transient expression of glucoraphanin biosynthetic genes from *A. thaliana* (Mikkelsen et al., 2010). As side-products, we obtained fivefold to eightfold higher levels of chain-elongated leucine-derived glucosinolates, not found in the native plant. Here, we investigated two different strategies to improve engineering of the methionine chain elongation part of the glucoraphanin pathway in *N. benthamiana*: (1) coexpression of the large subunit (*LSU1*) of the heterodimeric isopropylmalate isomerase and (2) coexpression of *BAT5* transporter for efficient transfer of intermediates across the chloroplast membrane. We succeeded in raising dihomomethionine (DHM) levels to a maximum of 432 nmol g^−1^ fresh weight that is equivalent to a ninefold increase compared to the highest production of this intermediate, as previously reported (Mikkelsen et al., 2010). The increased DHM production without increasing leucine-derived side-product levels provides new metabolic engineering strategies for improved glucoraphanin production in a heterologous host.

## Introduction

Plants are the source of an immense diversity of natural compounds, many of which are of high value as medicine or health-promoting agents. Often these compounds are difficult or impossible to produce by chemical synthesis, and extraction from plants is the only source.

Epidemiological studies strongly indicate that dietary consumption of cruciferous vegetables (e.g., broccoli) is correlated with reduced risk of the developing cancer (Verkerk et al., 2009). These and other health-promoting effects have been associated with glucosinolates, natural products characteristic to the Brassicales order, which include vegetables such as broccoli and cabbages and the model plant *Arabidopsis thaliana* (Halkier and Gershenzon, 2006).

Substantial attention has been given to particularly the glucosinolate glucoraphanin that is present in broccoli, as it is generally thought to be the major bioactive compound associated with the cancer-preventive effects of broccoli (Traka and Mithen, 2009; Kensler et al., 2013). A recent human intervention study showed that diets with glucoraphanin-enriched broccoli resulted in retuning of cellular processes in the mitochondria to a basal level that is critical for maintaining a healthy metabolic balance (Armah et al., 2013). The health-promoting effects have resulted in a strong desire to increase the intake of glucoraphanin. The current market is based on products with unreliable amounts of glucoraphanin, if any at all. The latter has primed an interest to engineer the production of glucoraphanin into a heterologous host to obtain a stable, rich source of this product and enable intake of well-defined doses for dietary and pharmaceutical applications.

As a prerequisite for pathway engineering, all glucoraphanin biosynthetic genes have been identified in the model plant *A. thaliana* (Sonderby et al., 2010). Previously, we have engineered the six glucosinolate core pathway genes of simple indolyl- and benzyl glucosinolate-derived directly from the protein amino acids tryptophan and phenylalanine into the non-cruciferous plant *Nicotiana benthamiana* (Geu-Flores et al., 2009; Pfalz et al., 2011), and for indolylglucosinolates also into yeast (Mikkelsen et al., 2012). Engineering of the complex glucoraphanin pathway presents additional challenges as it consists of 12 biosynthetic enzymes, which are partitioned between the chloroplast and the cytosol (Halkier and Gershenzon, 2006; Sonderby et al., 2010).

**Figure 1:**
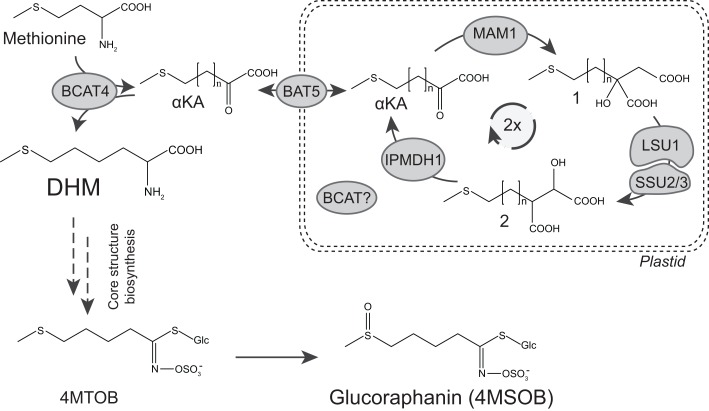
**Biosynthetic pathway of dihomomethionine and glucoraphanin in *Arabidopsis***. Glucoraphanin biosynthesis consists of three steps: methionine conversion to dihomomethionine (DHM) by two cycles in the chain elongation machinery. Followed by DHM conversion into 4-methylthiobutyl glucosinolate (4MTOB) by the core structure pathway, and finally conversion of 4MTOB into 4-methylsulfinyl GLS (4MSOB), commonly known as glucoraphanin. Structures for intermediates of methionine chain elongation are depicted with DHM as the desired intermediate for the formation of glucoraphanin. αKA = α-keto acid, methylthio malate intermediates after condensation (1) and after isomerization (2). *n* = number of additional methylene groups in the methionine side chain as a result of the condensation reaction in the chain elongation cycle: *n* = 1 for methionine, *n* = 2 and *n* = 3 for intermediates from the first and second side-chain elongation cycle, respectively.

Briefly, biosynthesis of glucoraphanin can be divided into three major parts (Figure 1). First, methionine is transaminated into a α-keto acid (α-KA) by a cytosolic branched-chain aminotransferase (BCAT4) (Schuster et al., 2006). This α-KA then enters the chloroplast where it undergoes side-chain elongation. The carbon side chain is elongated by a condensation reaction catalyzed by methylthioalkylmalate synthase (MAM) (Textor et al., 2004), followed by isomerization and oxidative decarboxylation catalyzed by an isopropylmalate isomerase (IPMI) and an isopropylmalate dehydrogenase (IPMDH), respectively (He et al., 2010, 2011). Following two cycles of chain elongation dihomomethionine (DHM) is formed, which subsequently is converted by the cytosolic, ER-associated core structure pathway to 4-methylthiobutyl (4MTOB) glucosinolate (GLS) (Figure 1), which finally is *S*-oxygenated to 4-methylsulfinylbutyl (4MSOB) glucosinolate, commonly known as glucoraphanin (Figure 1) (Sonderby et al., 2010).

The feasibility of metabolic engineering the complex glucoraphanin pathway was recently shown by transient expression of 10 biosynthetic genes in *N. benthamiana* (Mikkelsen et al., 2010). Though formation of ~50 nmol g^−1^ fresh weight (fw) of the chain-elongated methionine-derived glucoraphanin was detected, fivefold to eightfold more of chain-elongated leucine-derived glucosinolates were detected. The latter does not accumulate in the native *Arabidopsis* but have been observed under conditions when *MAM3* in the methionine chain elongation pathway was overexpressed using the 35S promoter (Field et al., 2004). Evolutionarily, the recursive methionine chain elongation pathway has evolved from the non-recursive valine to leucine chain elongation pathway in primary metabolism (Field et al., 2004; Textor et al., 2004; Schuster et al., 2006; Binder et al., 2007; He et al., 2010; de Kraker and Gershenzon, 2011). The ability to accumulate chain-elongated leucine-derived glucosinolates upon engineering in tobacco supports the proposed promiscuity of enzymes in specialized metabolism compared with those in primary metabolism (Weng and Noel, 2012). It also indicates that the native plant has evolved mechanism(s) to prevent the formation of these leucine-derived side-products.

Homologs are described for all genes in methionine chain elongation. Coexpression analysis and knockout mutants have helped to identify specific roles of the different homologs. MAM1 was identified as the enzyme catalyzing two condensation reactions (Textor et al., 2004). For IPMDH, three homologs are known of which IPMDH1 was recently reported to be the best candidate for methionine chain elongation, whereas IPMDH2 and IPMDH3 were involved in leucine biosynthesis (LeuC) (He et al., 2009, 2011, 2013). Nevertheless, IPMDH3 was previously shown to be functional in metabolic engineering of DHM (Mikkelsen et al., 2010). The isomerization reaction catalyzed by IPMI has yet another level of complexity. IPMI is a heterodimeric enzyme consisting of a single large subunit (LSU1) that forms a catalytic active enzyme with either of three small subunits (SSU1/2/3). The small subunits define in which pathway IPMI is active. SSU2 and SSU3 are generally associated with methionine chain elongation and SSU1 with LeuC (He et al., 2010). Nevertheless, it was recently hypothesized that SSU1 was actively involved in the first two cycles of methionine chain elongation (Imhof et al., 2014). Transport of α-keto acids (formed initially by cytosolic BCAT4 and after each cycle by the chloroplastic IPMDH enzyme) across the chloroplast membranes was suggested to be performed by the bile acid transporter 5 (BAT5) (Gigolashvili et al., 2009). This was based on *bat5* knockout mutants showing a 50% reduction in methionine-derived, aliphatic glucosinolates and transport of α-keto acids into the chloroplast was impaired.

Toward our goal to establish high glucoraphanin production in a heterologous host, optimization of DHM production is essential. General means to enhance product formation in pathway engineering projects include screening for lacking enzymes, enzymes with improved properties (i.e., substrate specificity, kinetics), alleviating catalytic bottlenecks and increasing flux through the pathway, and taking compartmentalization into account (Heinig et al., 2013). Here, we report optimization of the production of the glucoraphanin precursor DHM in *N. benthamiana*. As reference for comparison, we use the highest-producing gene combination, as previously reported. The increase is obtained by optimizing the combination of biosynthetic genes used. Moreover, we provide additional evidence that BAT5 is the transporter for α-keto acids across the chloroplast membrane system.

## Materials and Methods

### Plant Material

*Nicotiana benthamiana* plants were grown in small pots of 5.5 cm diameter in a green house at 24°C (day) and 18°C (night) with 50–60% humidity for ~3–4 weeks (to four to six leaves stage).

### Cloning and Transformation

All genes were cloned into a USER compatible version of pCambia3300^1^ plasmid by USER cloning (Nour-Eldin et al., 2006; Bitinaite et al., 2007; Geu-Flores et al., 2007). In brief, coding sequences of individual genes were amplified with single Uracil containing primers that were compatible with the USER ready-made plasmid. For PCR primers, see Table S1 in Supplementary Material. PCR products were purified (QIAquick PCR Purification Kit, Qiagen, Hilden, Germany), and 1–5 μL of purified PCR product were subsequently mixed with 1 μL of plasmid. The volume was adjusted to 10 μL and after addition of 1 μL USER Enzyme (NEB, Ipswich, MA, USA), the mix was incubated at 37 and 25°C for 30 min each. Two microliters of the USER cloning mix were added to 60 μl of chemical competent *E. coli* DHB10 (NEB, Ipswich, MA, USA) cells by heat shock. Briefly, 10 min on ice, 90 s 42°C followed by 2 min on ice. Cells were incubated for 60 min at 37°C after addition of 250 μL LB media. Subsequently, 100 μL were plated on LB agar plates containing kanamycin (50 μg mL^−1^). Correct gene insertions were verified by sequencing (Macrogen Europe, Amsterdam, Netherlands). Constructs with correct insertions were subsequently transformed into *Agrobacterium tumefaciens* strain pGV3850 by electroporation (2 mm cuvette, 2.5 kV, 400 Ω, and 25 μF) in a Bio-Rad GenePulser (Bio-Rad, Hercules, CA, USA). One milliliter YEP media was added, and cells were incubated for 3 h at 28°C with shaking. Subsequently, 150 μL were plated on YEP agar plates containing antibiotics (30 μg mL^−1^ rifampicin and 50 μg mL^−1^ kanamycin).

### Transient Expression by Infiltration of *Nicotiana benthamiana*

Overnight cultures of *A. tumefaciens* carrying the different gene constructs were grown in 10 mL YEP media (containing 50 μg mL^−1^ kanamycin and 30 μg mL^−1^ rifampicin) at 28°C and 220 rpm. Overnight cultures were harvested by centrifugation at 20°C for 15 min at 4500 × *g*. The cell pellets were resuspended in infiltration buffer (10 mM MgCl_2_, 10 mM MES, pH 5.6) containing 100 μM acetosyringone (3,5-dimethoxy-4-hydroxyacetophenone, Sigma-Aldrich, Steinheim, Germany) and shaken at 150 rpm for 1–2 h at room temperature prior to plant infiltration. Cell densities for all cultures was adjusted to OD_600_ ≈ 0.21–0.25, which resulted in a final concentration for each individual construct of OD_600_ ≈ 0.03 for experiments with chain elongation enzymes only and OD_600_ ≈ 0.015 for experiments, which included chain elongation and core structure enzymes. These low OD were sufficient to ensure efficient transformation while keeping the stress levels for *N. benthamiana* leaves low. In all experiments, the suppressor protein p19 was included to reduce silencing effects (Voinnet et al., 2003). For each individual, experiment two to three leaves of four *N. benthamiana* plants (3–4 weeks old) were infiltrated with the different combinations of *A. tumefaciens* cultures harboring the different gene constructs. A maximum of seven different *A. tumefaciens* cultures were mixed. The volume of combinations with fewer than seven constructs was adjusted by addition of the respective amount of infiltration buffer.

### Plant Material Harvesting and Sample Preparation

Plant material was harvested 5 days after infiltration with *A. tumefaciens*. From each leaf, four leaf disks of 1 cm diameter were harvested from infiltrated areas and weighed. Amino acids were extracted with 400 μL of 85% methanol containing norleucine (10 μM) as internal standard (IS). Amino acid concentrations were determined by comparison to 13C,15N-labeled algal amino acids described below.

### Amino Acid Analysis by LC-MS

The resulting extract was diluted in a ratio of 1:10 (v:v) in water containing the 13C,15N-labeled amino acid mix (Isotec, Miamisburg, OH, USA). Amino acids in the diluted extracts were directly analyzed by LC-MS/MS. The analysis method was modified from a protocol described by Jander et al. (2004). Chromatography was performed on an Agilent 1200 HPLC system (Agilent Technologies, Boeblingen, Germany). Separation was achieved on a Zorbax Eclipse XDB-C18 column (50 mm × 4.6 mm, 1.8 μm, Agilent Technologies, Germany). Formic acid (0.05%) in water and acetonitrile were employed as mobile phases A and B, respectively. The elution profile was 0–1 min, 3% B in A; 1–2.7 min, 3–100% B in A; 2.7–3 min 100% B, 3.1–6 min 3% B in A. The mobile phase flow rate was 1.1 mL/min. The column temperature was maintained at 25°C. The liquid chromatography was coupled to an API 5000 tandem mass spectrometer (AB Sciex, Darmstadt, Germany) equipped with a Turbospray ion source operated in positive ionization mode. The instrument parameters were optimized by infusion experiments with pure standards (amino acid standard mix, Fluka, St. Louis, MO, USA). The ionspray voltage was maintained at 5500 eV. The turbo gas temperature was set at 700°C. Nebulizing gas was set at 70 psi, curtain gas at 35 psi, heating gas at 70 psi, and collision gas at 2 psi. Multiple reaction monitoring (MRM) was used to monitor analyte parent ion → product ion: MRMs were chosen as in Jander et al. (2004) except for Arg (*m*/*z* 175 → 70) and Lys (*m*/*z* 147 → 84). In addition, MRMs for homomethionine (HM, *m*/*z* 164 → 118), DHM (*m*/*z* 178 → 132), and *S*-adenosylmethionine (SAM, *m*/*z* 399 → 136). The chain-elongated leucine products homo-leucine (HL, *m*/*z* 146 → 100), dihomo-leucine (DHL, *m*/*z* 160 → 114), and trihomo-leucine (THL, *m*/*z* 174 → 128) were also monitored, but exact quantification was not possible for DHL and THL due to lack of reference standards. Values for DHL and THL are calculated based on the assumption of an equal response factor of 1 compared to 13C,15N-labeled phenylalanine due to their similar behavior in fragmentation and ionization compared to leucine and HL. Detailed values for mass transitions can be found in Table S2 in Supplementary Material. Both Q1 and Q3 quadrupoles were maintained at unit resolution. Analyst 1.5 software (AB Sciex, Darmstadt, Germany) was used for data acquisition and processing. Linearity in ionization efficiencies were verified by analyzing dilution series of standard mixtures (amino acid standard mix, Fluka + Gln, Asn, and Trp, also Fluka). All samples were spiked with 13C,15N-labeled amino acids (algal amino acids 13C,15N, Isotec, Miamisburg, OH, USA) at a concentration of 10 μg of the mix per milliliter. The concentration of the individual labeled amino acids in the mix had been determined by classical HPLC–fluorescence detection analysis after pre-column derivatization with ortho-phthalaldehyde-mercaptoethanol using external standard curves made from standard mixtures (amino acid standard mix, Fluka + Gln, Asn, and Trp, also Fluka). Individual amino acids in the sample were quantified by the respective 13C,15N-labeled amino acid IS, except for tryptophan, and asparagin: tryptophan was quantified using 13C,15N-Phe applying a response factor of 0.42, asparagin was quantified using 13C,15N-Asp applying a response factor of 1.0.

### Statistical Analysis

Statistical analysis was performed with the SigmaPlot 12.0 statistics package (Systat Software, San Jose, CA, USA).

### Accession Numbers

Sequence data from this article can be found *via* the TAIR database^2^ under the AGI locus identifiers: *BCAT4* (At3g19710), *RBSC1A* (At1g67090), *BAT5* (At4g12030), *MAM1* (At5g23010), *IPMI-LSU1* (AT4g13430), *IPMI-SSU1* (At2g43090), *IPMI-SSU2* (At2g43100), *IPMI-SSU3* (At3g58990), *IPMDH1* (At5g14200), and *IPMDH3* (At1g31180).

## Results and Discussion

In this study, we identified a new combination of genes for methionine chain elongation that produced the highest level of DHM in *N. benthamiana*. Additionally, we measured how these optimizations affected the formation of chain-elongated leucine-derived side-products.

### Definition of a Reference Value for Optimization of DHM Production

**Figure 2:**
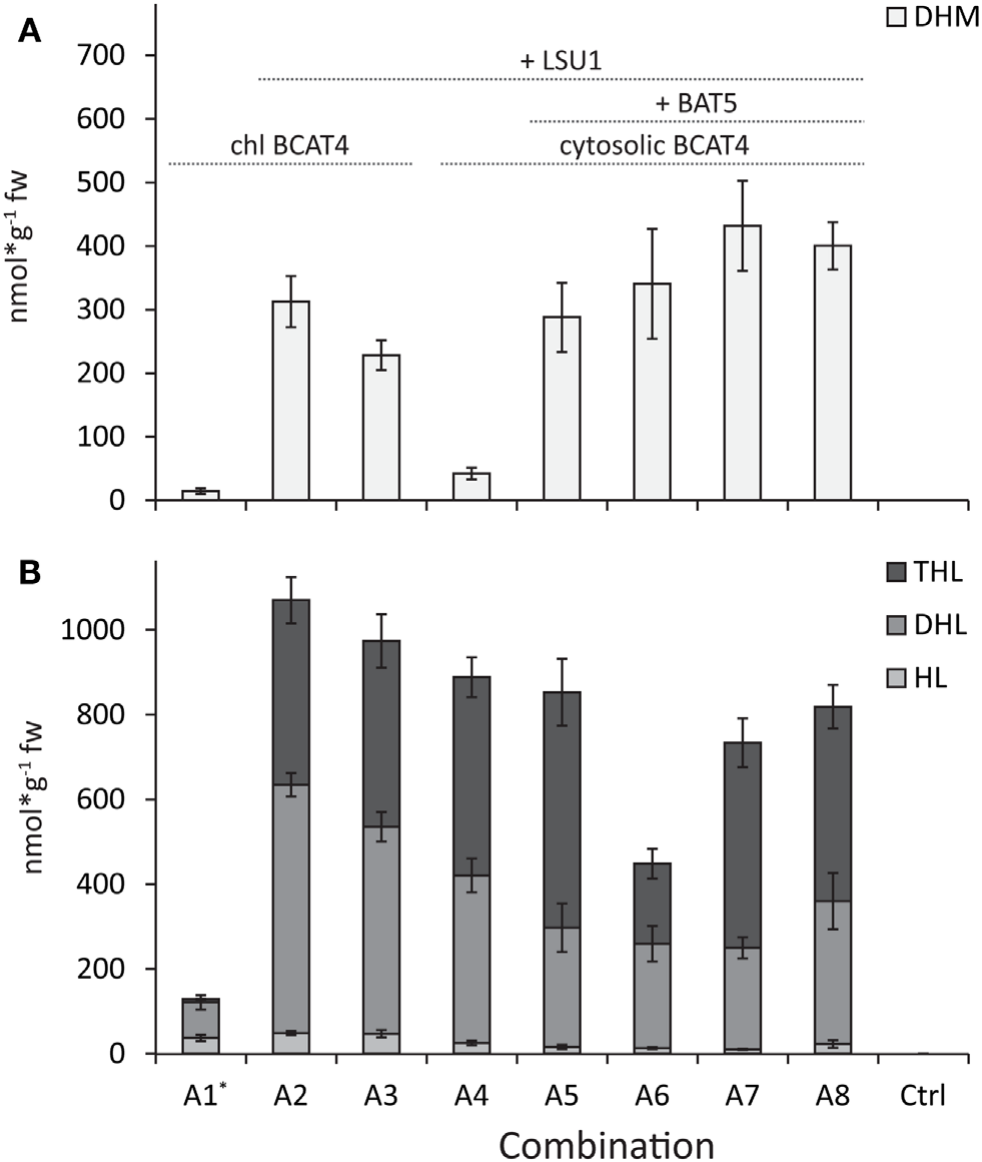
**Comparison of production of DHM and chain-elongated leucine-derived products**. **(A)** DHM levels. **(B)** Levels of homo-leucine (HL), dihomo-leucine (DHL), and trihomo-leucine (THL). BCAT4 is relocalized to the chloroplast (chl BCAT4) in combinations A1–A3. The transporter protein BAT5 is coexpressed in combinations A5–A8 and the large subunit (LSU1) of IPMI is coexpressed in all combinations except A1. Ctrl represents non-infiltrated. Chl BCAT4 = BCAT4 with signal peptide for relocation to chloroplast, +LSU1 = combinations where LSU1 was coexpressed, and +BAT5 = combinations where BAT5 was coexpressed. Data are represented as mean ± SEM in nanomole per gram fresh weight (*N* = 8).

**Figure 3:**
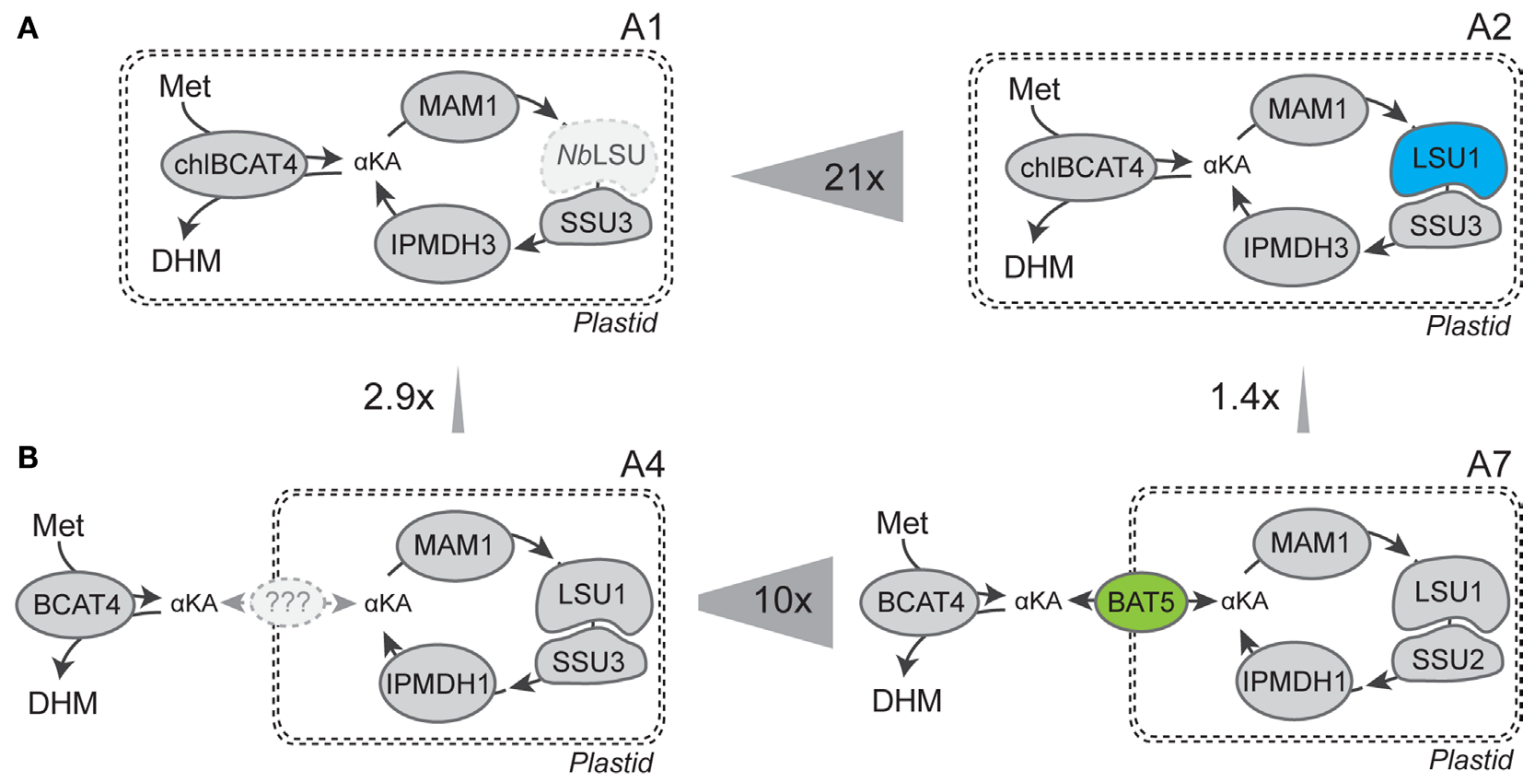
**Graphical comparison of combinations with most significant increases in DHM production**. **(A)** *Nicotiana benthamiana* LSU (*Nb*LSU) can complement *Arabidopsis* IPMI small subunit (SSU3) into a functional heterodimer, and co-expression of *Arabidopsis* IPMI large subunit (LSU1) increased DHM production by 21-fold. **(B)** BAT5 is an efficient transporter for translocation of intermediates between the cytosolic BCAT4 and the chloroplast-localized part of methionine chain elongation and increased DHM production by 10-fold.

Toward our goal of optimizing DHM production by transient expression experiments in *N. benthamiana*, we choose as reference a gene combination identical the highest-producing gene combination, as previously reported (Mikkelsen et al., 2010). In this study, genes were expressed from multi-gene constructs with two or three genes separated by 2A sequences (Mikkelsen et al., 2010). However, as we in the current study would compare multiple gene combinations, we expressed all genes from single gene constructs as this enabled us to freely combine individual genes. The previously reported gene combination for highest DHM production included a chloroplast-localized *BCAT4* together with *MAM1*, *IMPI–SSU3*, and *IPMDH3* and resulted in the formation of 51.4 ± 20.8 nmol g^−1^ fw (Mikkelsen et al., 2010). When we expressed the same genes individually, we obtained only 14.6 ± 4.4 nmol g^−1^ fw, which was used as reference value in this study (Table 1; Figures 2A and 3A). Several parameters can account for the discrepancy between the values published by Mikkelsen et al. (2010) and the present study. Using single gene constructs increases the number of *Agrobacterium* strains that need to be mixed for coexpression. It has also been reported that constructs containing 2A sequences for self-processing of multi-gene constructs can result in incomplete cleavage and formation of fusion proteins, which can influence the outcome of metabolic engineering from transient expression in plants (Burén et al., 2012). Other potential reasons include differences in growth conditions for the tobacco plants and differences in detection and quantification of the individual compounds by LC-MS between the two studies. In combination, the experimental and technical differences do not allow for a direct comparison of the DHM production. All calculations are based on the DHM amounts produced in the reference gene combination of the present study, which previously resulted in the highest DHM production.

**Table 1:** **Optimization of DHM production in *N. benthamiana***.

Combination	Genes	DHM	
A1 = Reference^a^	*chlBCAT4*, *MAM1*, *SSU3*, *IPMDH3*	14.6	(±4.4)
A2	*chlBCAT4*, *MAM1*, *LSU1*, *SSU3*, *IPMDH3*	312.6	(±40.2)
A3	*chlBCAT4*, *MAM1*, *LSU1*, *SSU3*, *IPMDH1*	228.5	(±23.5)
A4	*BCAT4*, *MAM1*, *LSU1*, *SSU3*, *IPMDH1*	41.9	(±9.2)
A5	*BCAT4*, *BAT5*, *MAM1*, *LSU1*, *SSU3*, *IPMDH1*	315.0	(±54.8)
A6	*BCAT4*, *BAT5*, *MAM1*, *LSU1*, *SSU1*, *IPMDH1*	340.6	(±86.5)
A7	*BCAT4*, *BAT5*, *MAM1*, *LSU1*, *SSU2*, *IPMDH1*	432.2	(±70.8)
A8	*BCAT4*, *BAT5*, *MAM1*, *LSU1*, *SSU1*, *SSU3*, *IPMDH1*	400.4	(±37.3)
Ctrl	Non-infiltrated	n.d.	

*Data are represented as mean ± SEM in nanomole per gram fresh weight (*N* = 8)*.

*DHM, dihomomethionine; fw, fresh weight; chl, chloroplastic signal peptide; BCAT4, branched-chain aminotransferase 4; MAM1, methylthioalkylmalate synthase 1; LSU1, large subunit of isopropylmalate isomerase (IPMI); SSU, small subunit of IPMI; IPMDH, isopropylmalate dehydrogenase; Ctrl, control*.

*^a^Reference to highest-producing gene combination previously reported (Mikkelsen et al., 2010) with 51.4 nmol DHM g^−1^ fw. For values of all amino acids, see also Table S3 in Supplementary Material*.

### Coexpression of *Arabidopsis IPMI-LSU1* is Essential for Efficient Formation of DHM by the Methionine Chain Elongation Pathway

In the heterodimeric isopropylmalate synthase (IPMI), the large subunit (LSU1) forms a functional enzyme with one of three small subunits (SSU1–3) catalyzing the isomerization step in the methionine and valine (to leucine) chain elongation machinery (Knill et al., 2009). Previously, it was shown that endogenous LSU of tobacco was able to substitute for the *A. thaliana* LSU1 (that was not included in the gene combination) and form a functional IPMI enzyme with *A. thaliana* SSUs that resulted in production of DHM (Mikkelsen et al., 2010). Here, we show that inclusion of *Arabidopsis*’ *LSU1* in the gene combination resulted in a 21-fold increase of DHM production (Table 1; Figures 2A and 3A, combinations A1 and A2). Coexpression of *A. thaliana* LSU1 probably has alleviated a bottleneck and thus increased flux through the pathway. Another possibility could be better interaction between the two *A. thaliana* subunits in comparison to a heterodimer formed from *N. benthamiana* LSU and *A. thaliana* SSU. However, the high amino acid sequence identity of ~95% between *A. thaliana* LSU1 and *Nicotiana sylvestris* LSU, a close relative of *N. benthamiana*, suggests that functionality is not impaired. Recently, it was also demonstrated that *A. thaliana* SSUs to a certain extend can complement *E. coli* knockout mutants lacking the respective SSU homolog in LeuC (Imhof et al., 2014). Therefore, the increased production of DHM is most likely a result of a better ratio between large subunit to small subunit and the formation of a higher number of catalytically active IMPI heterodimers as a result of coexpressing *LSU1* under the control of the same CaMV35S promoter as the other constructs.

### Choice of Small Subunit in IPMI Has No Significant Influence on DHM Production

It has been reported that the three small subunits (SSU1/2/3) define in which pathway IPMI is active, with SSU2 and SSU3 being associated with methionine chain elongation and SSU1 with LeuC (He et al., 2010). More recently, it was hypothesized that SSU1 was active in the first two cycles of methionine chain elongation and that either SSU2 or SSU3 catalyzes the formation of the longer chain-elongated products (Imhof et al., 2014). Interestingly, we detected no significant difference in DHM production when we coexpressed the individual small subunits of *IPMI* with the rest of the methionine chain elongation machinery (Table 1, combinations A5–A7). Furthermore, coexpression of *SSU1* with SSU3 did not result in significantly higher production of DHM (Table 1, combination A8). Coexpression of *SSU2* and *SSU3* was previously shown to have no positive effect on DHM production (Mikkelsen et al., 2010). Nevertheless, our data show that SSU1 has the ability to support the first two rounds of methionine chain elongation. At this point, it remains unclear if SSU1 catalyzes the first round(s) of methionine chain elongation in native *A. thaliana* plants and whether either SSU2 or SSU3 catalyzes the formation of the longer chain-elongated products, as previously suggested (Imhof et al., 2014). A previously observed sevenfold difference in DHM production between coexpression of *SSU2* and *SSU3* (Mikkelsen et al., 2010) was not observed. This was insofar puzzling as differences in the present study were not as dramatic and not significant for the different products upon coexpression of either of the three *SSU*s together with *LSU1* (Figure 2; Table 1, combinations A5–A7). One possible explanation could be that coexpression of *IPMI–LSU1* has alleviated effects that might be related to functional differences between the three SSUs. And even though *A. thaliana* IPMI can complement *E. coli* mutant strains deficient for the respective homologs in LeuC (*LeuC* and *LeuD*) (He et al., 2010; Imhof et al., 2014), it cannot be excluded that there are functional differences when catalyzing reactions in methionine chain elongation. Finally, it still remains unclear whether functional differences and the involvement of the different SSUs in either leucine biosynthesis or methionine chain elongation are the result of structural differences in the active sites or due to differences in temporal and spatial expression within different tissues in the plant (He et al., 2010; Imhof et al., 2014).

### Coexpression of the BAT5 Transporter Facilitates DHM Production with Cytosolic BCAT4

Bile acid transporter 5 has been proposed to translocate substrates and/or products of methionine chain elongation across the chloroplast membranes (Gigolashvili et al., 2009). The α-keto acid product of BCAT4 was proposed to be the substrate for import into the chloroplast by BAT5 as *bat5* knockout mutants show 50% reduced levels of aliphatic glucosinolates (including 4MSOB and 4MTB) and transport of α-keto acids into the chloroplast was impaired (Gigolashvili et al., 2009). As BAT5 may be critical for translocating the chain elongation products out of the chloroplast, we investigated whether inclusion of BAT5 together with cytosolic BCAT4 improves DHM production.

In experiments where *BCAT4* was expressed in the cytosol without coexpression of *BAT5*, a massive reduction in DHM production to only 41.9 (±9.2) nmol g^−1^ fw was observed compared to chloroplast-localized BCAT4 (Table 1; Figures 2A and 3A). This demonstrates that although methionine chain elongation is a heterologous pathway, *N. benthamiana* still has the ability to transport α-keto acid intermediates at a low level. DHM production was fully restored upon coexpression of *BAT5* with the cytosolic *BCAT4* (Figure 3B). DHM levels were higher than in combinations with chloroplast-localized BCAT4, though not significantly higher (Table 1). It is not known whether cytosolic BCAT4 (or other chloroplast-localized BCATs) are involved in transaminating the final chain-elongated methionine product, and whether a chain-elongated α-keto acid is substrate for export out of the chloroplast by BAT5. Nevertheless, the fact that DHM accumulated to such high levels indicated that BCAT4 may also transaminate the chain-elongated α-keto acids into the respective chain-elongated methionine and that BAT5 is an antiporter for the different chain-lengths α-keto acids.

### Differences in DHM Production with IPMDH3 Compared to IPMDH1

Three homologs exist for IPMDH, of which IPMDH3 was used for in the previous report for engineering DHM (Mikkelsen et al., 2010). A recent study linking association by coexpression suggested that IPMDH1 was the key player in methionine chain elongation while IPMDH2 and IPMDH3 were involved in LeuC (He et al., 2013). When we compared inclusion of *IPMDH1* or *IPMDH3* together with the other chain elongation genes, we observed that the production of DHM was always higher with *IPMDH3* though never significantly higher (Table 1). The variation from transient expression in tobacco made it impossible to identify significant differences. Nevertheless, association by coexpression in *Arabidopsis*, as previously reported (He et al., 2013), does not exclude enzymatic promiscuity (Weng and Noel, 2012) of the different IPMDH enzymes. This is also supported by the fact that there was no significant difference detected between the three IPMI small subunits (see above).

### Presence of LSU1 Has Major Effects on Formation of Leucine-Derived Side-Products but is Independent of BCAT4 Localization

Previously, when the approximately fivefold to eightfold higher levels of leucine-derived glucosinolates were monitored (Mikkelsen et al., 2010), it was not possible to differentiate if both leucine and isoleucine were taken as substrates by the chain elongation machinery. Here, we confirmed by UHPLC-MS analysis that only leucine and not isoleucine is taken as substrate (Mirza et al., 2016). Optimization of the gene combination for DHM production also affected the formation of chain-elongated leucine-derived products (Figure 2). Similar to DHM production, chain-elongated leucine-derived products [homo-leucine (HL), dihomo-leucine (DHL), and the newly detected trihomo-leucine (THL)] drastically increased by including LSU1 together with the other chain elongation genes (Figure 2). In contrast to DHM, leucine-derived products were formed in similar amounts independent of whether *BCAT4* was expressed in the cytosol or the chloroplast, except for combination A1 where *LSU1* was not coexpressed (Figure 2, combinations A1 and A4; Table S3 in Supplementary Material). Especially, the formation of DHL was higher when BCAT4 was localized to the chloroplast (Figure 2, combinations A2 and A3). Interestingly, formation of leucine-derived products – especially the longer chain-elongated products DHL and THL – was reduced in the gene combination when *SSU1* (A6) was coexpressed rather than *SSU2* (A7) or *SSU3* (A5) (Figure 2B). Also, THL was only found in combinations where *LSU1* was coexpressed, which may be related to the overall lower production in this combination or a reduced ability of tobacco LSU to support three rounds of leucine chain elongation. THL had not been described previously as a side-product from engineering of methionine chain elongation in tobacco (Mikkelsen et al., 2010) and neither from metabolic engineering of DHM in *E. coli* (Mirza et al., 2016).

### Increased DHM Formation Considerably Improves DHM to Side-Product Ratio

Increased DHM formation had positive effects on the ratio of DHM to leucine-derived products. When the ratios were calculated for the sum of leucine-derived products (HL + DHL + THL) compared to DHM in the individual experiments, we detected 8.8-fold more leucine-derived products compared to DHM in the reference combination A1 as previously reported for this combination (Mikkelsen et al., 2010). All other combinations showed a more favorable ratio of DHM to chain-elongated leucine-derived products. The best ratios with almost equal amounts were found in combinations A6 and A7 (DHM:HL/DHL/THL 0.8:1) containing the complete set of genes and compartmenatlization (Figures 2 and 3; Table S4 in Supplementary Material). The amounts of leucine-derived products remained largely unchanged throughout our experiments. Mainly DHL and the newly detected THL contributed to a large extent to the high amounts of leucine-derived products. The latter was drastically reduced in the gene combination including SSU1. Interestingly, we detected similar amounts of leucine-derived products in the combination without BAT5 (A4), which suggests that leucine α-keto acids are present and taken up by the methionine chain elongation machinery. A similar effect was seen in experiments without coexpression of *BCAT4* (data not shown). Therefore, other solutions for further reduction of leucine-derived products may include exchange or mutation of single enzymes in the methionine chain elongation to increase affinity toward methionine and away from leucine.

In summary, our experiments provide new insights toward improving engineering of DHM production by transient expression in *N. benthamiana*. Here, we demonstrated a substantial 30-fold increase in production of DHM (432 nmol g^−1^ fw) compared to the highest-producing gene combination previously reported and a not less impressive 9-fold increase compared to the previously highest reported DHM production levels (Mikkelsen et al., 2010). Simultaneously, the amounts of leucine-derived side-products were substantially reduced especially by re-establishing the compartmentalized organization of the methionine chain elongation in the transient expression host system. In conclusion, the optimized gene combination for production of DHM consists of five (or six) genes: *BCAT4* (*BAT5*), *MAM1*, *LSU1*, *SSU1*, and *IPMDH1*. *BAT5* is only necessary if the methionine chain elongation is expressed in plants or other chloroplast-containing organisms, such as microalgae, while in the case of a microbial host, the transport step can be omitted. Our results provide important insights for optimizing the engineering of glucoraphanin production in a heterologous host. Especially, the fact that the separation of the transamination step from the chain elongation itself improved the production of DHM mainly by reducing formation of leucine-derived side-products has implications on metabolic engineering in microbial host organisms where such spatial separation is difficult to establish. This implies that other techniques might be required to create a similar level of spatial separation between the two parts of the chain elongation pathway. This could include creation of fusion proteins or coexpression of chaperons to create microenvironments that also could increase flux through the pathway. In addition, it should be considered to engineer pathway enzymes in a way to increase substrate specificity toward methionine and the corresponding intermediates. These steps should be considered before adding another level of complexity by engineering the core structure biosynthetic pathway on top of the methionine chain elongation pathway to ultimately create an expression system for sustainable production of glucoraphanin.

## Author Contributions

CC cloned the constructs for DHM biosynthesis, planned and conducted the experiments, prepared the samples for LC-MS analysis for DHM production. NM conducted initial experiments, contributed to the discussion of results and the manuscript. MR performed analysis of amino acid and DHM production and suggested analytical improvements. JG contributed to the experimental design and made improvements to the manuscript. BH was involved in discussions about the experimental setup and results. CC and BH wrote the manuscript based on a draft written by CC.

## Conflict of Interest Statement

The authors declare that the research was conducted in the absence of any commercial or financial relationships that could be construed as a potential conflict of interest.

## Footnotes

^1^http://www.cambia.org/

^2^http://www.arabidopsis.org

## References

[B1] Armah C. N. Traka M. H. Dainty J. R. Defernez M. Janssens A. Leung W. (2013). A diet rich in high-glucoraphanin broccoli interacts with genotype to reduce discordance in plasma metabolite profiles by modulating mitochondrial function. Am. J. Clin. Nutr. 98, 712–722. 10.3945/ajcn.113.065235 23964055 PMC3743733

[B2] Binder S. Knill T. Schuster J. (2007). Branched-chain amino acid metabolism in higher plants. Physiol. Plant. 129, 68–78. 10.1111/j.1399-3054.2006.00800.x

[B3] Bitinaite J. Rubino M. Varma K. H. Schildkraut I. Vaisvila R. Vaiskunaite R. (2007). USER™ friendly DNA engineering and cloning method by uracil excision. Nucleic Acids Res. 35, 1992–2002. 10.1093/nar/gkm041 17341463 PMC1874603

[B4] Burén S. Ortega-Villasante C. Ötvös K. Samuelsson G. Bakó L. Villarejo A. (2012). Use of the foot-and-mouth disease virus 2A peptide co-expression system to study intracellular protein trafficking in *Arabidopsis*. PLoS ONE 7:e51973. 10.1371/journal.pone.0051973 23251667 PMC3522588

[B5] de Kraker J. W. Gershenzon J. (2011). From amino acid to glucosinolate biosynthesis: protein sequence changes in the evolution of methylthioalkylmalate synthase in *Arabidopsis*. Plant Cell 23, 38–53. 10.1105/tpc.110.079269 21205930 PMC3051243

[B6] Field B. Cardon G. Traka M. Botterman J. Vancanneyt G. Mithen R. (2004). Glucosinolate and amino acid biosynthesis in *Arabidopsis*. Plant Physiol. 135, 828–839. 10.1104/pp.104.039347 15155874 PMC514118

[B7] Geu-Flores F. Nour-Eldin H. H. Nielsen M. T. Halkier B. A. (2007). USER fusion: a rapid and efficient method for simultaneous fusion and cloning of multiple PCR products. Nucleic Acids Res. 35, e55. 10.1093/nar/gkm106 17389646 PMC1874642

[B8] Geu-Flores F. Olsen C. Halkier B. (2009). Towards engineering glucosinolates into non-cruciferous plants. Planta 229, 261–270. 10.1007/s00425-008-0825-y 18830705

[B9] Gigolashvili T. Yatusevich R. Rollwitz I. Humphry M. Gershenzon J. Flügge U.-I. (2009). The plastidic bile acid transporter 5 is required for the biosynthesis of methionine-derived glucosinolates in *Arabidopsis thaliana*. Plant Cell 21, 1813–1829. 10.1105/tpc.109.066399 19542295 PMC2714935

[B10] Halkier B. A. Gershenzon J. (2006). Biology and biochemistry of glucosinolates. Annu. Rev. Plant Biol. 57, 303–333. 10.1146/annurev.arplant.57.032905.105228 16669764

[B11] He Y. Chen B. Pang Q. Strul J. M. Chen S. (2010). Functional specification of *Arabidopsis* isopropylmalate isomerases in glucosinolate and leucine biosynthesis. Plant Cell Physiol. 51, 1480–1487. 10.1093/pcp/pcq113 20663849

[B12] He Y. Dai S. Dufresne C. P. Zhu N. Pang Q. Chen S. (2013). Integrated proteomics and metabolomics of *Arabidopsis* acclimation to gene-dosage dependent perturbation of isopropylmalate dehydrogenases. PLoS ONE 8:e57118. 10.1371/journal.pone.0057118 23533573 PMC3606340

[B13] He Y. Galant A. Pang Q. Strul J. M. Balogun S. F. Jez J. M. (2011). Structural and functional evolution of isopropylmalate dehydrogenases in the leucine and glucosinolate pathways of *Arabidopsis thaliana*. J. Biol. Chem. 286, 28794–28801. 10.1074/jbc.M111.262519 21697089 PMC3190687

[B14] He Y. Mawhinney T. P. Preuss M. L. Schroeder A. C. Chen B. Abraham L. (2009). A redox-active isopropylmalate dehydrogenase functions in the biosynthesis of glucosinolates and leucine in *Arabidopsis*. Plant J. 60, 679–690. 10.1111/j.1365-313X.2009.03990.x 19674406

[B15] Heinig U. Gutensohn M. Dudareva N. Aharoni A. (2013). The challenges of cellular compartmentalization in plant metabolic engineering. Curr. Opin. Biotechnol. 24, 239–246. 10.1016/j.copbio.2012.11.006 23246154

[B16] Imhof J. Huber F. Reichelt M. Gershenzon J. Wiegreffe C. Lächler K. (2014). The small subunit 1 of the *Arabidopsis* isopropylmalate isomerase is required for normal growth and development and the early stages of glucosinolate formation. PLoS ONE 9:e91071. 10.1371/journal.pone.0091071 24608865 PMC3946710

[B17] Jander G. Norris S. R. Joshi V. Fraga M. Rugg A. Yu S. (2004). Application of a high-throughput HPLC-MS/MS assay to *Arabidopsis* mutant screening; evidence that threonine aldolase plays a role in seed nutritional quality. Plant J. 39, 465–475. 10.1111/j.1365-313X.2004.02140.x 15255874

[B18] Kensler T. Egner P. Agyeman A. Visvanathan K. Groopman J. Chen J.-G. (2013). “Keap1-Nrf2 signaling: a target for cancer prevention by sulforaphane,” in Natural Products in Cancer Prevention and Therapy, eds Pezzuto J. M. Suh N. (Berlin; Heidelberg: Springer), 163–177. 10.1007/128_2012_339 PMC3553557 22752583

[B19] Knill T. Reichelt M. Paetz C. Gershenzon J. Binder S. (2009). *Arabidopsis thaliana* encodes a bacterial-type heterodimeric isopropylmalate isomerase involved in both Leu biosynthesis and the Met chain elongation pathway of glucosinolate formation. Plant Mol. Biol. 71, 227–239. 10.1007/s11103-009-9519-5 19597944 PMC2729411

[B20] Mikkelsen M. D. Buron L. D. Salomonsen B. Olsen C. E. Hansen B. G. Mortensen U. H. (2012). Microbial production of indolylglucosinolate through engineering of a multi-gene pathway in a versatile yeast expression platform. Metab. Eng. 14, 104–111. 10.1016/j.ymben.2012.01.006 22326477

[B21] Mikkelsen M. D. Olsen C. E. Halkier B. A. (2010). Production of the cancer-preventive glucoraphanin in tobacco. Mol. Plant. 3, 751–759. 10.1093/mp/ssq020 20457641

[B22] Mirza N. Crocoll C. Erik Olsen C. Ann Halkier B. (2016). Engineering of methionine chain elongation part of glucoraphanin pathway in *E. coli*. Metab. Eng. 35, 31–37. 10.1016/j.ymben.2015.09.012 26410451

[B23] Nour-Eldin H. H. Hansen B. G. Nørholm M. H. H. Jensen J. K. Halkier B. A. (2006). Advancing uracil-excision based cloning towards an ideal technique for cloning PCR fragments. Nucleic Acids Res. 34, e122. 10.1093/nar/gkl635 17000637 PMC1635280

[B24] Pfalz M. Mikkelsen M. D. Bednarek P. Olsen C. E. Halkier B. A. Kroymann J. (2011). Metabolic engineering in *Nicotiana benthamiana* reveals key enzyme functions in *Arabidopsis* indole glucosinolate modification. Plant Cell 23, 716–729. 10.1105/tpc.110.081711 21317374 PMC3077789

[B25] Schuster J. Knill T. Reichelt M. Gershenzon J. Binder S. (2006). Branched-chain aminotransferase4 is part of the chain elongation pathway in the biosynthesis of methionine-derived glucosinolates in *Arabidopsis*. Plant Cell 18, 2664–2679. 10.1105/tpc.105.039339 17056707 PMC1626624

[B26] Sonderby I. E. Geu-Flores F. Halkier B. A. (2010). Biosynthesis of glucosinolates – gene discovery and beyond. Trends Plant Sci. 15, 283–290. 10.1016/j.tplants.2010.02.005 20303821

[B27] Textor S. Bartram S. Kroymann J. Falk K. L. Hick A. Pickett J. A. (2004). Biosynthesis of methionine-derived glucosinolates in *Arabidopsis thaliana*: recombinant expression and characterization of methylthioalkylmalate synthase, the condensing enzyme of the chain-elongation cycle. Planta 218, 1026–1035. 10.1007/s00425-003-1184-3 14740211

[B28] Traka M. Mithen R. (2009). Glucosinolates, isothiocyanates and human health. Phytochem. Rev. 8, 269–282. 10.1007/s11101-008-9103-7

[B29] Verkerk R. Schreiner M. Krumbein A. Ciska E. Holst B. Rowland I. (2009). Glucosinolates in *Brassica* vegetables: the influence of the food supply chain on intake, bioavailability and human health. Mol. Nutr. Food Res. 53, S219–S265. 10.1002/mnfr.200800065 19035553

[B30] Voinnet O. Rivas S. Mestre P. Baulcombe D. (2003). An enhanced transient expression system in plants based on suppression of gene silencing by the p19 protein of tomato bushy stunt virus. Plant J. 33, 949–956. 10.1046/j.1365-313X.2003.01676.x 12609035

[B31] Weng J.-K. Noel J. P. (2012). The remarkable pliability and promiscuity of specialized metabolism. Cold Spring Harb. Symp. Quant. Biol. 77, 309–320. 10.1101/sqb.2012.77.014787 23269558

