# Supplementary material for: Optimization of Engineered Production of the Glucoraphanin Precursor Dihomomethionine in Nicotiana benthamiana
Source: Front Bioeng Biotechnol. 2016 Feb 16;4:14. doi: 10.3389/fbioe.2016.00014 (PMC4754535; doi:10.3389/fbioe.2016.00014)
Supplement: Supplementary file 1 [file Table_1.DOCX]

**Supplemental Information**

**Table S1: Primers for USER cloning of expression constructs**

| **Gene**  (AGI code) | **Primer name** | **Sequence 5’- 3’** | **Remarks** |
| --- | --- | --- | --- |
| BCAT4 | BCAT4uF | GGCTTAAU**atg**gctccttctgcgcaa |  |
| (At3g19710) | BCAT4uR | GGTTTAAU**tca**gccctggcggtcaatctc |  |
| chlBCAT4 | rbcSuF | GGCTTAAU**atg**gcttcctctatgctctctt | Signal peptide from rubisco small subunit 1A (At1g67090) fused to BCAT4 |
|  | rbcS-BCAT4PhuR | AGCCATAAGGUaagagagagtctcaaacttc |  |
|  | rbcS-BCAT4PhuF | accttatggcUCCTTCTGCGCAACC |  |
|  | BCAT4uR | GGTTTAAU**tca**gccctggcggtcaatctc |  |
| BAT5 | BAT5uF | GGCTTAAU**atg**ggtgtgatatctccgactg |  |
| (At4g12030) | BAT5uR | GGTTTAAU**cta**ctcctttctgtttttccatatggt |  |
| MAM1 | MAM1uF | GGCTTAAU**atg**gcttcatcgcttctgacatcttc |  |
| (At5g23010) | MAM1uR | GGTTTAAU**tta**cacattcgatgaaacctgagga |  |
| IPMI-LSU1 | IPMI-LSU1uF | GGCTTAAU**atg**gcttctgttatctcttcctct |  |
| (AT4g13430) | IPMI-LSU1uR | GGTTTAAU**cta**ctgcaagaactcccttgg |  |
| IPMI-SSU1 | IPMI-SSU1uF | GGCTTAAU**atg**gcggcttctctgca |  |
| (At2g43090) | IPMI-SSU1uR | GGTTTAAU**tca**agcagcagcagatgg |  |
| IPMI-SSU2 | IPMI-SSU2uF | GGCTTAAU**atg**gcgtattctcttcctacatttc |  |
| (At2g43100) | IPMI-SSU2uR | GGTTTAAU**tta**agctaatgatggaatcatt |  |
| IPMI-SSU3 | IPMI-SSU3uF | GGCTTAAU**atg**gcgacttctcagcaatt |  |
| (At3g58990) | IPMI-SSU3uR | GGTTTAAUtcaagcagaaggaatcatgc |  |
| IPMDH1 | IPMDH1uF | GGCTTAAU**atg**gcggcgtttttgcaaac |  |
| (At5g14200) | IPMDH1uR | GGTTTAAU**tta**aacagtagctggaactttggatt |  |
| IPMDH3 | IPMDH3uF | GGCTTAAU**atg**gcggcgtttttgcaaactaac |  |
| (At1g31180) | IPMDH3uR | GGTTTAAU**tta**aacaggaactttggagtccac |  |

Primer overhangs for USER cloning in UPPERCASE letters; Start and Stop codons are **underlined**.
