# Supplementary material for: Optimization of Engineered Production of the Glucoraphanin Precursor Dihomomethionine in Nicotiana benthamiana
Source: Front Bioeng Biotechnol. 2016 Feb 16;4:14. doi: 10.3389/fbioe.2016.00014 (PMC4754535; doi:10.3389/fbioe.2016.00014)
Supplement: Supplementary file 2 [file Table_2.DOCX]

**Supplemental Information**

**Table S2: MRMs for amino acids and glucosinolates determined by LC-MS/MS**

| **amino acid** | **Q1** | **Q3** | **DP** | **CE** | **internal standard** | **Q1** | **Q3** | **response**  **factor** |
| --- | --- | --- | --- | --- | --- | --- | --- | --- |
| Ala | 90.1 | 44.1 | 51 | 17 | 13C,15N-Ala | 94.1 | 47.1 |  |
| Ser | 106.0 | 60.1 | 61 | 15 | 13C,15N-Ser | 110.0 | 63.1 |  |
| Pro | 116.1 | 70.0 | 61 | 19 | 13C,15N-Pro | 122.1 | 75.0 |  |
| Val | 118.1 | 72.2 | 56 | 13 | 13C,15N-Val | 124.1 | 77.2 |  |
| Thr | 120.1 | 74.2 | 56 | 13 | 13C,15N-Thr | 125.1 | 78.2 |  |
| Ile | 132.2 | 86.1 | 56 | 13 | 13C,15N-Ile | 139.2 | 92.1 |  |
| Leu | 132.2 | 86.1 | 56 | 13 | 13C,15N-Leu | 139.2 | 92.1 |  |
| Norleu | 132.2 | 86.1 | 56 | 13 | 13C,15N-Leu |  |  | 1.00 |
| Asp | 134.1 | 74.1 | 56 | 19 | 13C,15N-Asp | 139.1 | 77.1 |  |
| Glu | 148.1 | 102.1 | 56 | 15 | 13C,15N-Glu | 154.1 | 107.1 |  |
| Met | 150.2 | 104.1 | 51 | 13 | 13C,15N-Met | 156.2 | 109.1 |  |
| His | 156.2 | 110.1 | 61 | 17 | 13C,15N-His | 165.2 | 118.1 |  |
| Phe | 166.2 | 120.2 | 56 | 17 | 13C,15N-Phe | 176.2 | 129.2 |  |
| Arg | 175.1 | 70.1 | 66 | 31 | 13C,15N-Arg | 185.1 | 75.1 |  |
| Tyr | 182.1 | 136.2 | 56 | 17 | 13C,15N-Tyr | 192.1 | 145.2 |  |
| Asn | 133.1 | 74.1 | 56 | 21 | 13C,15N-Asp | 139.1 | 77.1 | 1.00 |
| Gln | 147.1 | 130.0 | 61 | 13 | 13C,15N-Gln | 154.1 | 136.0 |  |
| Trp | 205.2 | 188.1 | 56 | 13 | 13C,15N-Phe |  |  | 0.42 |
| Lys | 147.1 | 84.1 | 61 | 23 | 13C,15N-Lys | 155.1 | 90.1 |  |
| HM | 164.2 | 118.1 | 51 | 13 | 13C,15N-Tyr |  |  | 2.40 |
| DHM | 178.2 | 132.1 | 51 | 13 | 13C,15N-Phe |  |  | 1.05 |
|  |  |  |  |  |  |  |  |  |
| HL | 146.2 | 100.1 | 56 | 13 | 13C,15N-Phe |  |  | 1.00* |
| DHL | 160.2 | 114.1 | 56 | 13 | 13C,15N-Phe |  |  | 1.00* |
| THL | 174.2 | 128.1 | 56 | 13 | 13C,15N-Phe |  |  | 1.00* |

*Response factors for HL, DHL and THL were assumed to be 1.00.
