# Supplementary material for: Optimization of Engineered Production of the Glucoraphanin Precursor Dihomomethionine in Nicotiana benthamiana
Source: Front Bioeng Biotechnol. 2016 Feb 16;4:14. doi: 10.3389/fbioe.2016.00014 (PMC4754535; doi:10.3389/fbioe.2016.00014)
Supplement: Supplementary file 3 [file Table_3.DOCX]

**Supplemental Information**

**Table S3: Values for amino acids and chain-elongated methionine and leucine products from experimental series A (Tables 1 and 2).** Data are represented as mean ± SEM in nmol*g^-1^ fresh weight, N=8.

| **Amino**  **acid** | **Combination** | | | | | | | | |
| --- | --- | --- | --- | --- | --- | --- | --- | --- | --- |
|  | **A1** | **A2** | **A3** | **A4** | **A5** | **A6** | **A7** | **A8** | **Ctrl** |
| **Ala** | 8173.1 | 8689.3 | 7192.0 | 7548.6 | 10409.9 | 9112.5 | 9851.0 | 11121.1 | 7015.7 |
| ±SE | 841.7 | 855.3 | 716.8 | 990.4 | 655.8 | 1026.5 | 580.6 | 620.0 | 866.2 |
| **Ser** | 6570.9 | 4361.1 | 4752.7 | 3824.6 | 6079.7 | 4939.9 | 4436.6 | 5345.6 | 12101.2 |
| ±SE | 54.5 | 552.3 | 464.4 | 612.4 | 770.8 | 584.9 | 350.2 | 1177.4 | 1275.9 |
| **Pro** | 5428.1 | 2945.0 | 3378.3 | 2402.3 | 5036.7 | 1528.1 | 2954.2 | 2662.1 | 7763.0 |
| ±SE | 1289.8 | 403.5 | 1103.3 | 717.0 | 1219.8 | 213.0 | 545.4 | 634.7 | 3002.4 |
| **Val** | 1433.6 | 1714.9 | 1992.1 | 1644.7 | 2214.9 | 1642.5 | 1652.3 | 2329.7 | 920.6 |
| ±SE | 145.0 | 198.9 | 321.8 | 232.8 | 278.9 | 196.2 | 107.3 | 543.1 | 109.0 |
| **Thr** | 5074.5 | 5740.0 | 5021.0 | 4141.3 | 7617.1 | 4985.2 | 5629.2 | 6962.2 | 3833.6 |
| ±SE | 548.7 | 586.3 | 404.5 | 617.6 | 925.3 | 465.1 | 531.6 | 1239.6 | 642.0 |
| **Ile** | 1479.0 | 1983.2 | 2215.9 | 1963.4 | 3506.1 | 1765.7 | 1873.6 | 2710.6 | 446.4 |
| ±SE | 298.5 | 386.5 | 493.4 | 422.3 | 597.9 | 339.0 | 225.4 | 687.4 | 47.7 |
| **Leu** | 863.3 | 1034.9 | 1179.7 | 929.0 | 1379.6 | 890.3 | 876.6 | 1532.9 | 437.5 |
| ±SE | 109.2 | 117.4 | 234.7 | 178.1 | 194.7 | 112.5 | 49.1 | 429.4 | 43.8 |
| **Asp** | 4258.9 | 4505.5 | 4068.3 | 3305.2 | 3878.5 | 4248.5 | 4482.9 | 4190.7 | 8343.9 |
| ±SE | 627.5 | 450.6 | 316.6 | 407.7 | 727.3 | 572.8 | 421.7 | 399.0 | 1643.1 |
| **Glu** | 12154.9 | 12549.0 | 12196.4 | 9439.5 | 14718.9 | 14350.1 | 15809.6 | 14192.5 | 27002.3 |
| ±SE | 1440.5 | 1134.7 | 1016.9 | 1169.0 | 1730.0 | 930.3 | 1087.7 | 824.7 | 5440.0 |
| **Met** | 108.0 | 126.1 | 139.7 | 175.0 | 163.3 | 121.6 | 134.1 | 169.8 | 137.1 |
| ±SE | 14.2 | 9.0 | 14.9 | 17.0 | 11.9 | 10.7 | 10.6 | 23.3 | 25.7 |
| **Phe** | 1744.7 | 2109.9 | 2224.8 | 2022.6 | 2420.1 | 1643.0 | 1828.3 | 2291.4 | 1107.4 |
| ±SE | 146.0 | 110.6 | 220.1 | 309.0 | 329.3 | 151.2 | 178.6 | 493.4 | 68.7 |
| **Arg** | 1327.7 | 1709.8 | 1849.3 | 1617.4 | 2017.7 | 1544.9 | 1968.0 | 2489.7 | 230.5 |
| ±SE | 186.9 | 147.8 | 204.3 | 236.4 | 67.6 | 133.5 | 167.2 | 545.9 | 39.7 |
| **Tyr** | 1023.0 | 1307.3 | 1312.6 | 969.2 | 1710.7 | 1072.2 | 1278.3 | 1447.9 | 133.5 |
| ±SE | 168.2 | 164.8 | 204.3 | 161.7 | 181.3 | 164.4 | 165.4 | 119.7 | 12.6 |
| **Trp** | 281.3 | 327.8 | 395.0 | 331.7 | 724.1 | 309.3 | 442.1 | 460.6 | 21.9 |
| ±SE | 40.1 | 42.4 | 60.8 | 54.8 | 105.9 | 60.8 | 57.3 | 91.3 | 3.6 |
| **Asn** | 3323.7 | 3228.7 | 5077.8 | 4875.5 | 3609.1 | 3735.1 | 3009.5 | 7926.6 | 2775.3 |
| ±SE | 486.4 | 700.8 | 2487.4 | 3334.9 | 1158.7 | 1032.6 | 322.5 | 4286.4 | 448.7 |
| **Gln** | 162784.2 | 145767.8 | 137855.2 | 110141.1 | 121203.9 | 95921.4 | 137675.3 | 138678.8 | 95553.1 |
| ±SE | 23140.5 | 8165.4 | 22616.6 | 20622.1 | 17607.3 | 16146.7 | 14205.4 | 13188.7 | 14425.4 |
| **Lys** | 962.5 | 1513.1 | 1618.4 | 1472.5 | 2155.7 | 1506.9 | 1370.0 | 2148.6 | 209.8 |
| ±SE | 184.9 | 258.3 | 339.1 | 428.6 | 471.9 | 310.7 | 225.0 | 582.4 | 17.7 |
| **DHM** | 14.6 | 312.6 | 228.5 | 41.9 | 288.1 | 340.6 | 432.2 | 400.4 | n.d. |
| ±SE | 4.4 | 40.2 | 23.5 | 9.2 | 54.6 | 86.5 | 70.8 | 37.3 |  |
| **HL** | 37.4 | 48.9 | 47.2 | 25.3 | 15.9 | 13.1 | 10.6 | 23.3 | 0.1 |
| ±SE | 7.5 | 5.0 | 8.4 | 5.3 | 4.9 | 2.2 | 1.3 | 9.2 | 0.0 |
| **DHL** | 84.1 | 585.9 | 488.7 | 395.4 | 281.4 | 246.8 | 239.5 | 336.7 | 1.5 |
| ±SE | 17.2 | 27.7 | 34.8 | 39.8 | 56.9 | 41.8 | 25.1 | 66.3 | 0.8 |
| **THL** | 6.8 | 435.4 | 438.3 | 468.0 | 555.7 | 188.6 | 483.9 | 458.8 | 4.0 |
| ±SE | 1.2 | 54.8 | 63.0 | 46.9 | 78.8 | 35.2 | 57.7 | 51.3 | 2.4 |
