# Supplementary material for: Optimization of Engineered Production of the Glucoraphanin Precursor Dihomomethionine in Nicotiana benthamiana
Source: Front Bioeng Biotechnol. 2016 Feb 16;4:14. doi: 10.3389/fbioe.2016.00014 (PMC4754535; doi:10.3389/fbioe.2016.00014)
Supplement: Supplementary file 4 [file Table_4.DOCX]

**Supplemental Information**

**Table S4 Ratios between DHL and leucine-derived products (HL/DHL/THL).**

|  | **Combination** | | | | | | | | |
| --- | --- | --- | --- | --- | --- | --- | --- | --- | --- |
|  | **A1** | **A2** | **A3** | **A4** | **A5** | **A6** | **A7** | **A8** |  |
| Sum HL/DHL/THL | 128,3 | 1070,2 | 974,2 | 888,7 | 853 | 448,5 | 734 | 818,8 |  |
| DHM:HL/DHL/THL | 0,1 | 0,3 | 0,2 | 0,0 | 0,3 | **0,8** | **0,6** | 0,5 |  |
