# Supplementary material for: Optimization of Engineered Production of the Glucoraphanin Precursor Dihomomethionine in Nicotiana benthamiana
Source: Front Bioeng Biotechnol. 2016 Feb 16;4:14. doi: 10.3389/fbioe.2016.00014 (PMC4754535; doi:10.3389/fbioe.2016.00014)
Supplement: Supplementary file 5 [file Table_5.DOCX]

**Supplemental Information**

**Table S5: Gene combinations used for optimization of DHM production in *Nicotiana benthamiana*.** Results for gene combinations are found in Table 2 and S3.

|  | **Gene** | | | | | | | | | | |
| --- | --- | --- | --- | --- | --- | --- | --- | --- | --- | --- | --- |
| **Gene**  **Combination** | **BCAT4** | **chlBCAT4** | **BAT5** | **MAM1** | **IMPI-LSU1** | **IPMI-SSU1** | **IPMI-SSU2** | **IPMI-SSU3** | **IPMDH1** | **IPMDH3** |  |
| **A1=Reference*** |  | x |  | x |  |  |  | x |  | x |  |
| **A2** |  | x |  | x | x |  |  | x |  | x |  |
| **A3** |  | x |  | x | x |  |  | x | x |  |  |
| **A4** | x |  |  | x | x |  |  | x | x |  |  |
| **A5** | x |  | x | x | x |  |  | x | x |  |  |
| **A6** | x |  | x | x | x | x |  |  | x |  |  |
| **A7** | x |  | x | x | x |  | x |  | x |  |  |
| **A8** | x |  | x | x | x | x |  | x | x |  |  |

*= highest producing gene combination previously reported ([Mikkelsen et al., 2010](#_ENREF_31)).
